# Systemic immune-inflammation index as a versatile biomarker in autoimmune disorders: insights from rheumatoid arthritis, lupus, and spondyloarthritis

**DOI:** 10.3389/fimmu.2025.1621209

**Published:** 2025-08-07

**Authors:** Yang Wu, Yupeng Huang, Yan Wu, Jianhong Sun, Qibing Xie, Geng Yin

**Affiliations:** ^1^ Department of General Practice, General Practice Medical Center, West China Hospital, Sichuan University, Chengdu, Sichuan, China; ^2^ Department of Rheumatology and Immunology, West China Hospital, Sichuan University, Chengdu, Sichuan, China; ^3^ Outpatient Department, West China Hospital, Sichuan University, Chengdu, Sichuan, China

**Keywords:** systemic immune-inflammation index, autoimmune diseases, rheumatoid arthritis, systemic lupus erythematosus, biomarkers of inflammation

## Abstract

Autoimmune diseases, such as systemic lupus erythematosus (SLE), rheumatoid arthritis (RA), systemic vasculitis, spondyloarthritis (SpA), including ankylosing spondylitis (AS) and psoriatic arthritis (PsA), are characterized by chronic immune activation and systemic inflammation. The systemic immune-inflammation index (SII), computed as platelet count × neutrophil count/lymphocyte count, is a promising biomarker that reflects both inflammatory burden and immune dysregulation. In RA, elevation of SII is correlated with disease activity score, response to TNF-α inhibitors, and reduced serum Klotho levels. In AS and PsA, the SII is associated with disease activity scores, musculoskeletal imaging findings, and treatment response. In SLE, the SII tracks global activity and predicts lupus nephritis and pregnancy outcomes, while further reflecting underlying features, such as lymphopenia, neutrophil extracellular trap formation, and platelet activation. The SII is also useful in vasculitis-related diseases, including Behçet’s syndrome and Kawasaki disease. In comparison to traditional markers such as CRP and ESR, the SII provides broader immune insights than routine hematologic data. SII is influenced by non-autoimmune factors, including malignancy and infection, which are often excluded from autoimmune studies, although significant in clinical interpretation. This review summarizes the latest evidence on the SII across autoimmune conditions. It also aims to outline the key limitations and future directions, including longitudinal validation, integration with emerging indices (e.g., the systemic inflammatory response index), and its role in multimodal disease monitoring.

## 1 Introduction

Autoimmune diseases are characterized by the presence of autoimmunity accompanied by a clinically identifiable pathology (1–3). These conditions affect approximately 7–9% of the global population (4). Systemic lupus erythematosus (SLE), rheumatoid arthritis (RA), spondyloarthritis (SpA), and systemic vasculitis are chronic immune-mediated disorders characterized by persistent inflammation and multiorgan involvement (5, 6).

Despite diagnostic and therapeutic advancements, there is a critical need for reliable, accessible, and dynamic biomarkers to guide disease management for treatment strategy optimization. In this context, the systemic immune-inflammation index (SII) has emerged as a promising marker, initially validated in vasculitis, oncology, and cardiovascular research, reflecting the systemic balance between inflammatory and immune responses (7–10).

The SII, initially developed in oncology and cardiovascular research, has demonstrated potential utility in autoimmune diseases. This mini review aims to critically evaluate the current evidence on the role of the SII in RA, SLE, SpA, and systemic vasculitides, highlight its potential as a diagnostic and prognostic biomarker, identify current limitations, and propose directions for future research.

## 2 Definition and clinical relevance of SII

The SII is a composite marker that incorporates three types of circulating immune cells: neutrophils, platelets, and lymphocytes. It is calculated using the following formula:


$$SII=\frac{\left(platelet\;count\;\times \;neutrophil\;count\right)}{lymphocyte\;count}$$

First introduced by Hu et al. (11) in 2014 as a prognostic marker for hepatocellular carcinoma (HCC), the SII has been associated with increased all-cause death rates across a range of disorders, including cancer, cardiovascular disease, and metabolic disorders. Additionally, several studies have suggested that the SII may serve as a useful index of disease progression in specific clinical contexts (12–16). Given its capacity to simultaneously reflect both proinflammatory and immunoregulatory dynamics, the SII has garnered increasing interest in the field of immunology.

## 3 Pathophysiological basis of SII in autoimmunity

Immune cells that constitute the SII, including neutrophils, lymphocytes, and platelets, are directly implicated in the pathogenesis of autoimmune diseases (17).

Neutrophils play a central role in initiating and amplifying inflammation through neutrophil extracellular trap (NET) formation, cytokine release, and direct tissue damage (18). In RA, neutrophils infiltrate synovial tissue and release proteolytic enzymes and reactive oxygen species, contributing to joint destruction (19, 20). In SLE, excess NETs expose the nuclear antigens and promote autoantibody formation (21).

Lymphocytes, particularly CD4 + T and B cells, are integral to the autoimmune pathophysiology. RA is predominantly associated with Th1- and Th17-mediated responses (22, 23), while SLE is characterized by aberrant B cell activation, autoantibody production, and impaired regulatory T cell function (24). Lymphocyte count serves as a surrogate marker of immune homeostasis disruption, and the opposing roles of regulatory T cells (Tregs) and pro-inflammatory subsets such as Th17 cells may obscure critical nuanced immunological dynamics (25–27). This limitation highlights the need for integrative biomarker frameworks that combine the SII with subset-specific immunophenotyping to more accurately reflect disease pathophysiology.

Platelets are traditionally regarded as hemostatic elements that are active participants in immune modulation. They interact with leukocytes, release inflammatory mediators, and contribute to endothelial dysfunction. In SLE, platelet activation is linked to vascular inflammation and thrombosis [9], while in RA, activated platelets promote synovial inflammation through cytokine release and immune cell recruitment (28, 29).

By integrating these three cellular parameters, the SII offers a more comprehensive assessment of the immune-inflammatory milieu than individual biomarkers. The balance between the regulatory (lymphocytes) and pro-inflammatory (neutrophils and platelets) elements captured by the SII may serve as an integrative marker of systemic immune dysregulation in autoimmune diseases. The applications of SII in autoimmune and inflammatory diseases are listed in **Table 1**.

**Table 1:** List of SII applications in autoimmune and inflammatory diseases.

Disease	Reference	Activity Assessment	Cut-off Value	Therapy Response	Key Findings and Remarks
RA	(38)	+	574.2	/	SII distinguished active disease from remission and controls, outperforming SIRI.
RA	(39)	+	305.6	/	SII was positively correlated with disease activity, and showed moderate diagnostic accuracy for active RA but poor sensitivity for remission detection.
RA	(41)	/	/	+	SII was among the strongest predictors of TNF-α inhibitor efficacy.
RA	(40)	/	578.25	/	Elevated ln-SII was independently linked to increased RA prevalence, with risk markedly rising above the threshold of 578.25.
RA	(42)	/	/	/	Higher SII quartiles were associated with progressively lower Klotho levels.
SLE	(76)	–	/	/	SII was elevated in SLE but showed weaker diagnostic value and no significant link to disease activity or renal involvement.
SLE	(77)	/	1612.6	/	First-trimester SII was significantly elevated in SLE pregnancies with adverse outcomes.
SLE	(75)	+	545.9	/	SII was an independent risk factor for lupus nephritis and moderately predictive of renal involvement.
SLE	(73)	+	415	/	SII reflected systemic inflammation and was associated with high disease activity and ANA positivity.
SLE	(71)	+	681.3	/	SII showed the strongest correlation with SLEDAI and the highest diagnostic performance (AUC = 0.930).
SLE	(78)	+	/	–	SII elevated in SLE but lacked independent prognostic value for flare or treatment response.
SLE	(72)	+	Excluded^a^	/	SII demonstrated high discriminative power for severe disease flares (AUC=0.963).
SLE	(74)	+	1348.4	/	SII was significantly associated with both SLE disease activity and renal involvement.
AS	(58)	+	513.2	/	SII outperformed traditional markers for AS activity assessment.
AS	(57)	–	/	/	SII reflects inflammation but not clinical activity or health status.
Psoriasis	(61)	–	/	+	SII was higher in patients comorbid psoriatic arthritis.
PsA	(62)	+	490	/	SII as independent marker for disease severity and PsA risk.
PsA	(63)	+	800	+	SII correlates with PsA activity, and practical for monitoring treatment response.
BS	(84)	+	552	/	SII showed strong discriminatory ability in distinguishing active from inactive BS.
BS	(86)	/	/	/	SII demonstrated strong ability to differentiate vascular involvement in BS (AUC=0.87).
BS	(85)	+	526.23		SII independently predicted active BS with a cut-off of 526.23.
MPO-AAV	(83)	–	2,136.45	/	SII ≥2,136.45 associated with significantly better cumulative renal survival.
KD	(87)	/	/	/	Elevated SII independently associated with CALs; AUC=0.789 for predicting coronary involvement at admission.
KD	(89)	+	1006.11	/	Post-IVIG SII independently predicted IVIG resistance (AUC=0.706).
KD	(88)	/	668.6	/	Lower SII independently associated with presence of CALs.
KD	(90)	/	2,209.66	+	SII was an independent risk factor for IVIG resistance (AUC=0.626)

SII, systemic immune-inflammation index; RA, rheumatoid arthritis; SLE, systemic lupus erythematosus; AS, ankylosing spondylitis; PsA, psoriatic arthritis; BS, Behçet’s syndrome; MPO-AAV, myeloperoxidase−anti−neutrophil cytoplasmic antibody−associated vasculitis; KD, Kawasaki disease; SLEDAI, SLE Disease Activity Index; CALs, coronary artery lesions.

a The reported SII cutoff value of 877,002.19 was excluded due to a clear internal inconsistency with hematological parameters, suggesting a probable miscalculation or unit mismatch. Only the AUC was retained to reflect the diagnostic insights of the study.

## 4 SII in RA

### 4.1 Background

RA is a chronic systemic autoimmune disorder characterized by persistent synovial inflammation, progressive joint destruction, and various extra-articular manifestations (30). Its complex pathogenesis involves disrupted bone remodeling due to an imbalance between osteoblast and osteoclast activity, synoviocyte hyperplasia, and immune dysregulation (31). At the cellular level, the aberrant activation of T cells, B cells, and macrophages drives chronic inflammation and tissue damage. This immune activation is accompanied by excessive secretion of pro-inflammatory cytokines such as tumor necrosis factor-alpha (TNF-α), interleukin-6 (IL-6), and interleukin-17 (IL-17) (24, 32, 33).

Emerging evidence suggests that impaired regulatory T cell function and the expansion of autoreactive T cells promote B cell hyperactivity and autoantibody production in RA (24, 34, 35). Dysfunctional immune homeostasis, marked by impaired T-cell regulation and excessive B-cell activity, plays a central role in perpetuating autoimmunity (36, 37). As RA progresses, systemic inflammation intensifies and can be quantitatively assessed using hematological indices. The neutrophil-to-lymphocyte ratio (NLR) and platelet-to-lymphocyte ratio (PLR) have shown greater sensitivity in reflecting inflammatory burden than individual cell counts (37). More comprehensively, the SII, which incorporates lymphocyte, neutrophil, and platelet counts, serves as an applicable composite index of the immune-inflammatory status in RA.

### 4.2 SII in the evaluation of disease activity and inflammation

The SII has emerged as a promising biomarker for systemic inflammation and immune cell dynamics. Thus in RA, the SII has shown value in assessing disease activity and inflammatory burden.

Satis et al. (38) reported that the SII levels were significantly higher in patients with RA compared to healthy controls (HCs), with a positive correlation between the SII values and disease activity. Active RA was associated with higher SII (702.25 ± 39.56) than remission (574.69 ± 34.72). Additional data showed mean SII levels of 666.42 ± 33.00 in patients with RA versus 596.71 ± 57.64 in HCs.

Supporting evidence from Choe et al. (39) demonstrated significant correlations between SII and composite disease activity scores, including the Disease Activity Score (DAS)28-ESR, DAS28-CRP, CDAI, and SDAI. SII values increased with disease severity, suggesting that it may be a sensitive cell-based index for tracking RA progression.

Population-based data support this association. A cross-sectional NHANES analysis (n = 37,604) (40) found that a higher SII was independently associated with an increased RA prevalence. Restricted cubic spline analysis revealed a non-linear dose–response relationship, with an inflection point at ln-SII = 6.36 (SII ≈ 578.25), beyond which RA risk increased sharply.

### 4.3 SII and therapeutic response in RA

Although RA remains the primary focus, the SII has demonstrated utility in predicting treatment response. In a retrospective study of 154 patients with RA treated with TNF-α inhibitors, Bai et al. (41) found that pre-treatment SII levels were significantly lower in responders than in non-responders. Among the tested inflammatory markers, the SII and lymphocyte count exhibited the strongest predictive value for therapeutic efficacy, outperforming conventional biomarkers such as CRP and RF. These findings support SII’s potential in stratifying patients for biological therapy and predicting outcomes.

### 4.4 Association with molecular markers: the Klotho link

Recent findings suggest that the SII may reflect not only immune activation, but also molecular alterations associated with aging and immune dysregulation. A study of 982 patients with RA from the NHANES 2007–2016 identified a remarkable inverse association between the SII and serum levels of soluble Klotho protein (42), and a significant inverse correlation was observed between the SII and serum Klotho protein—an anti-aging, anti-inflammatory molecule secreted mainly by the kidneys and parathyroid glands (43, 44). Serum Klotho concentrations quantified using ELISA showed consistent inverse correlations with SII across regression models, independent of age, comorbidities, or metabolic status (42).

Mechanistically, Klotho modulates immune and oxidative stress pathways. Experimental studies indicate that Klotho attenuates reactive oxygen species, suppresses pro-inflammatory cytokines (e.g., IL-6, TNF-α), and inhibits NF-κB signaling (45). It also influences the neutrophil activity, lymphocyte function, and platelet activation (46–48). In RA, where macrophage-driven inflammation contributes to joint destruction (36, 49), reduced Klotho levels may indicate the failure of endogenous anti-inflammatory regulation. The observed inverse trend between SII and serum Klotho suggests that SII may reflect not only immune cell imbalance but also the underlying immunosenescence and molecular stress.

### 4.5 Diagnostic value and cut-off determination

Several studies have evaluated the diagnostic utility of the SII and have proposed clinically relevant cutoff values. Satis et al. (38), reported an AUC of 0.643 for SII in distinguishing active RA, with sensitivity of 56.3% and specificity of 45.5% at a threshold of 574.20. In contrast, a study of female RA patients proposed a lower cutoff of 305.6, yielding 85% sensitivity and 42% specificity for distinguishing patients from healthy controls (39). These findings suggest that the SII may serve as an accessible and cost-effective adjunct for screening and disease monitoring, particularly when conventional biomarkers are inaccurate.

Another study comparing the SII and pan-immune-inflammation value (PIV) found that both indices were elevated in patients with active RA compared to those in remission or HCs. PIV outperformed the SII in distinguishing remission from health, suggesting that PIV may offer superior sensitivity in detecting low-grade inflammation (50, 51). Another ROC-based study confirmed the acceptable diagnostic performance of the SII in active disease but noted limited utility for remission detection (39).

### 4.6 Summary and clinical implications

Collectively, the current evidence highlights the clinical relevance of the SII in RA. It shows consistent correlation with composite disease activity indices (e.g., DAS28 and CDAI) (38, 39), enables stratification by inflammatory burden and treatment response (41), and reflects broader immunometabolic dysregulation, including Klotho deficiency (42). Proposed cutoffs—305.6 (sensitivity, 85%; specificity, 42%) (39) may assist in disease monitoring and risk classification. Satis et al. (38) reported an AUC of 0.643 for SII in distinguishing active RA, with sensitivity of 56.3% and specificity of 45.5% at a cutoff of 574.20. These findings collectively suggest that SII thresholds require careful optimization to maximize diagnostic utility.

However, this study has a few limitations. The SII’s discriminatory power diminishes in low-inflammatory or near-remission states. In these cases, novel indices, such as PIV, may provide better sensitivity. In support of this, ROC analyses showed that, while the SII retained fair accuracy in active RA, it was less effective for remission monitoring (39).

Future studies should validate the current cutoffs longitudinally and define SII dynamics across treatment courses. Integrating the SII with conventional markers (e.g., CRP and ESR) may enhance diagnostic precision, while a combination with emerging indices such as the systemic inflammation response index (SIRI) may expand its value in predicting RA-related complications, including interstitial lung disease and malignancy (52).

### 5 SII in AS and PsA

### 5.1 Background

AS and PsA are the two major subtypes of SpA that primarily affect the axial skeleton and peripheral joints (53). These diseases are frequently accompanied with systemic manifestations and comorbidities. Immunologically, both AS and PsA are driven by dysregulated immune responses and sustained inflammation, contributing to progressive joint damage, reduced mobility, and functional disability (54–56).

### 5.2 SII in disease activity assessment

Recent studies have evaluated the utility of SII in assessing disease activity in patients with AS and PsA. In a cross-sectional study involving 100 patients with SpA (including both AS and PsA), the median SII exceeded 600, indicating active systemic inflammation. Patients with high disease activity demonstrated significantly elevated SII levels, which were positively correlated with conventional inflammatory markers, such as ESR and CRP, supporting the potential of SII as an objective indicator of inflammatory burden in SpA (57).

Additional evidence was obtained from a retrospective study involving 136 patients with AS and 63 HCs, which identified the SII as the most effective single biomarker for differentiating active diseases. Using a cut-off value of 513.2, the SII achieved 86.84% sensitivity and 83.33% specificity for detecting active AS, outperforming conventional laboratory indices (58).

Furthermore, in a study assessing patients with combined axial and peripheral SpA, an increased SII was significantly associated with active disease, as defined by BASDAI > 4 and DAPSA > 14. Notably, SII strongly correlates with ultrasonography-detected synovitis in peripheral SpA, suggesting its value as a marker of both systemic and local inflammation (57).

### 5.3 SII and therapeutic response in psoriasis and PsA

Psoriasis, especially when accompanied by PsA, is increasingly being recognized as a systemic inflammatory condition that extends beyond cutaneous involvement (59). Biological therapies with anti-inflammatory mechanisms have demonstrated efficacy in managing skin and joint symptoms, systemic inflammation, and associated comorbidities (60).

In this therapeutic context, the SII has shown potential as a marker of treatment response. In a study of 220 patients with psoriasis, SII values significantly decreased after three months of biologic therapy (P< 0.001) (61). Subgroup analyses revealed significant reductions across several classes of biologic agents, including TNF-α inhibitors, IL-17 inhibitors, and IL-12/23 inhibitors, as well as with individual agents such as adalimumab, infliximab, ixekizumab, secukinumab etc. This suggests that the SII may serve as a reliable marker for monitoring therapeutic efficacy and predicting remission in patients with psoriasis and PsA.

Another study reported significantly higher SII in patients with PsA than in those with psoriasis. SII values were positively correlated with PASI scores and the presence of arthritis, further supporting its potential role as a prognostic marker for psoriatic disease (62). Moreover, the SII was significantly elevated in patients with moderate-to-severe PsA disease activity, as defined by the DAPSA scores. A proposed diagnostic cutoff of 800 yields high sensitivity and specificity for disease activity identification (63).

### 5.4 Summary and clinical implications

Collectively, the current evidence supports the clinical utility of the SII in assessing disease activity in AS and monitoring treatment responses in psoriasis and PsA. Its strong correlation with clinical indices and imaging findings, along with its association with PsA severity and progression, highlight its prognostic value. However, this study had several limitations. Disease-specific cutoffs have not yet been standardized, and their applicability across different SpA subtypes and disease stages requires further clarification. Future studies should prioritize longitudinal validation, integration of conventional markers and emerging immune signatures, and evaluation using multiparametric clinical algorithms (57).

## 6 SII in SLE

### 6.1 Background

SLE is a chronic multisystem autoimmune disorder characterized by alternating flares, remission, and dysregulation of the innate and adaptive immunity (64, 65). Clinically, SLE presents with heterogeneous manifestations involving the skin, joints, kidneys, central nervous system, and hematopoietic system (66, 67). The cellular components of SII are pathophysiologically relevant to SLE.

Neutrophils form NETs and exacerbate inflammation and tissue injury (68). Lymphopenia, a hallmark of active SLE, reflects T cell exhaustion and immune dysregulation, and lymphocyte counts and ratios serve as markers of immune disturbances and autoantibody-driven inflammation (69). Platelet activation contributes to the endothelial damage and inflammation. Elevated platelet counts or activity indicates heightened inflammation and may be correlated with disease activity and organ damage (70). Thus, SII may act as a composite indicator of disease burden and immune imbalance, especially during flares.

### 6.2 SII and global disease activity

In multiple cohorts, the SII was consistently correlated with global disease activity indices in SLE, particularly the SLE Disease Activity Index (SLEDAI or SLEDAI-2K). Akdogan et al. (71) reported a strong correlation between the SII and SLEDAI (r = 0.698, p = 0.01), with high discriminatory power for moderate-to-severe activity (AUC = 0.930, sensitivity 77%, specificity 76%). Predescu et al. (72) similarly identified the SII as a robust flare marker, with an AUC of 0.963 for severe flares. Baykal et al. (73) observed an elevated SII in pediatric patients with high disease activity, especially in ANA-positive cases.

### 6.3 Organ-specific associations: lupus nephritis and pregnancy outcomes

SII may reflect organ involvement, particularly in renal disease. Ergun et al. (74) showed a higher SII in patients with lupus nephritis than in those without proteinuria (p = 0.012), with correlations between both proteinuria and SLEDAI. Yang et al. confirmed that the SII independently predicted lupus nephritis (AUC = 0.6775) and correlated with SLEDAI-2K (r = 0.24, p< 0.05) (75). However, Özdemir et al. found no significant difference in the SII between patients with and without nephritis (76).

Sahin et al. demonstrated that an elevated first-trimester SII in pregnant patients with SLE was associated with adverse outcomes, including preterm birth and fetal loss. The optimal threshold (1612.6) yielded a sensitivity of 73.3% and specificity of 71.4% (77).

### 6.4 Treatment response monitoring

Longitudinal data on treatment-related SII changes are limited but promising. Gambichler et al. (78) observed that clinical and serological improvements were accompanied by a decline in the SII and rising lymphocyte counts, suggesting their potential utility in therapy monitoring. However, these findings are exploratory and require further validation.

### 6.5 Clinical applicability and limitations

The SII offers a rapid, noninvasive measure of systemic inflammation and has demonstrated utility in autoimmune conditions. However, its nonspecific nature is a limitation of this study. Taha et al. (79) reported elevated SII levels in patients with SLE, RA, and AS. Moreover, its components may vary with physiological changes or treatments, which complicates its interpretation.

Predescu et al. (72) reported a strong discriminative capacity of the SII for severe SLE flares (AUC=0.963), which aligned with the findings of Akdogan et al. (AUC=0.930) (71). However, the implausible SII cutoff (877,002.19)—reflects likely computational/unit errors given the incongruence with hematologic parameters (mean platelets: 246.413 ± 118.145×10³/µL, neutrophils: 3.818 ± 1.381×10³/µL, lymphocytes 1.591 ± 0.707×10³/µL). Consequently, while the AUC validates the SII’s diagnostic potential, the cutoff was excluded from the synthesis to prevent misinterpretation. This underscores the importance of a rigorous methodology for threshold derivation for the clinical translation of biomarkers.

Despite these challenges, the SII may complement conventional markers, such as anti-dsDNA and complement levels, particularly where access to immunologic assays is limited. Future work should define disease-specific cutoffs, track longitudinal changes, and assess the role of the SII in multiparametric models integrating clinical, serological, and imaging data.

## 7 Systemic immune-inflammation index in vasculitides

### 7.1 Background

Systemic vasculitides, including antineutrophil cytoplasmic antibody (ANCA)-associated vasculitis (AAV), Behçet’s syndrome (BS), and Kawasaki disease (KD), are complex inflammatory conditions characterized by vascular inflammation and multisystem involvement (80–82). Accurate biomarkers are essential for evaluating disease activity, organ involvement, and treatment responses. In vasculitis, where disease activity and vascular injury are often difficult to assess, the SII may offer additional diagnostic and prognostic value.

### 7.2 SII in disease activity assessment

A retrospective study of 190 Chinese patients with myeloperoxidase (MPO)-AAV found that the baseline SII was positively correlated with CRP and ESR, but not with the Birmingham Vasculitis Activity Score (BVAS). Interestingly, patients with higher SII values (≥2136.45) had a significantly lower risk of progression to end-stage renal disease (ESRD), suggesting a possible protective association (83).

Several studies have linked the SII to disease activity in BS. In a retrospective analysis, Tanacan et al. (84) reported a significantly higher SII in active-BS than in inactive-BS, with a proposed cutoff value of 552 × 10^9^. Similarly, Menteseoglu and Atakan (85) found that an elevated SII correlated with CRP level, ESR, NLR, and PLR, and was an independent predictor of active disease in regression analysis.

A large cohort study by Ulusoy et al. (86) showed a significantly higher SII in patients with BS and vascular involvement than in those with mucocutaneous or other phenotypes. The SII demonstrated a strong discriminatory capacity for vascular involvement (AUC = 0.87).

In KD, multiple studies have evaluated the role of the SII in predicting coronary artery lesions (CALs), with mixed results. Huang et al. (87) found a significant linear association between the SII and CALs at admission, with an AUC of 0.789.

In contrast, Yalcinkaya et al. (88) observed a lower SII in patients with KD and CALs and identified male sex, low prognostic nutritional index (PNI), and reduced SII as independent risk factors. Liu et al. (89) found that post-intravenous immunoglobulin (IVIG) SII levels were significantly higher in IVIG-resistant patients, although the baseline SII did not differ according to resistance status.

### 7.3 SII and therapeutic response in vasculitides

In addition to its role in disease activity, SII may help assess treatment response and prognosis. For MPO-AAV, Chen et al. (83) found that a higher baseline SII correlated with CRP level and ESR, but not BVAS, and was associated with improved renal survival and reduced ESRD risk (83).

In KD, an elevated baseline SII is associated with IVIG resistance. Yi et al. (90) reported an optimal cutoff of 2209.66 (AUC of 0.626) and identified the SII, SIRI, and PIV as independent predictors of non-responsiveness. Similarly, Liu et al. (89) observed a significantly higher post-treatment SII in IVIG-resistant patients (AUC = 0.706), indicating that the failure of the SII to decline may signal persistent inflammation and predict resistance.

### 7.4 Summary and clinical implications

Emerging data support the use of the SII across vasculitis subtypes. In AAV, elevated SII is correlated with inflammatory markers and predicts renal outcomes (83). In the BS, the SII distinguishes active from inactive disease (84, 85), correlates with inflammatory indices, and differentiates vascular from mucocutaneous forms with high accuracy (AUC of up to 0.87) (86). In KD, SII predicts CALs and IVIG resistance, reflecting disease severity and treatment response (87, 90).

Overall, the SII is a simple, accessible, and cost-effective index for evaluating inflammation and vascular involvement in vasculitis. However, disease-specific cutoffs and prospective validations are needed to refine their clinical utility alongside established biomarkers.

## 8 SII in oncology and infection: insights into comorbidities

The SII has been linked to the prognosis and clinicopathological features of various tumors (91). In gastric and lung cancers, reductions in the SII after neoadjuvant therapy were significantly associated with major pathological responses and favorable outcomes (92, 93). Meta-analyses of colorectal cancer have confirmed that an elevated SII is a robust predictor of poor overall and disease-free survival (94). Additionally, UK Biobank data showed that an elevated pre-diagnostic SII was associated with higher cancer incidence, particularly within one year of diagnosis, suggesting a possible paraneoplastic inflammatory response (95).

In ICU patients with sepsis, both low and high SII values were associated with a higher 28-day mortality, forming a J-shaped risk curve (96). In diabetes patients with odontogenic infections, SII showed a high predictive value for severe infections (97). The SII also correlated with pneumonia risk in patients with intracerebral hemorrhage, although the performance varied across inflammatory markers (98). Another study identified the SIRI and nutritional scores as independent predictors of pneumonia and in-hospital death in the same population (99). In children undergoing cardiopulmonary bypass, a lower early postoperative SII is associated with an increased risk of nosocomial infections (100).

These findings highlight the broad utility of SII in cancer and infections. Most studies have focused on autoimmune diseases such as RA, SLE, SpA, and vasculitis. As both conditions can elevate the SII independently of autoimmunity, their exclusion is methodologically appropriate but also highlights a clinical limitation. When using the SII to monitor autoimmune inflammation, clinicians should consider possible confounders, such as occult malignancy or subclinical infection.

## 9 Conclusion and perspectives

The SII shows potential as a composite biomarker of immune-inflammatory activity across autoimmune diseases, including RA, SLE, SpA, and vasculitides. By integrating neutrophil, platelet, and lymphocyte counts, the SII provides an accessible and cost-effective adjunct for screening and disease monitoring. However, several challenges remain to be resolved. The reported optimal SII cutoffs vary substantially (e.g., 305.60–578.25 in RA vs. 415.00–1612.60 in SLE), reflecting disease-specific pathophysiology, endpoint definitions (e.g., renal involvement vs. global activity), and statistical methodologies. Its limited specificity and modest accuracy, along with confounding from infections or malignancies and cutoff heterogeneity, warrant cautious interpretation and further validation in large, well-characterized cohorts.

The clinical value of the SII may be enhanced by combining it with conventional indices and emerging biomarkers, such as the SIRI, or molecular indicators, such as Klotho. Future studies should prioritize the standardization of disease activity definitions and analytical protocols to harmonize the SII cutoff derivation. A more integrated approach that incorporates clinical, imaging, and molecular data may improve personalized management of autoimmune diseases.

## References

[1] Davidson A Diamond B. Autoimmune diseases. N Engl J Med. (2001) 345:340–50. doi: 10.1056/NEJM200108023450506, PMID: 11484692

[2] Wang L Wang FS Gershwin ME. Human autoimmune diseases: a comprehensive update. J Intern Med. (2015) 278:369–95. doi: 10.1111/joim.12395, PMID: 26212387

[3] Hedrich CM. Shaping the spectrum - From autoinflammation to autoimmunity. Clin Immunol. (2016) 165:21–8. doi: 10.1016/j.clim.2016.03.002, PMID: 26948930

[4] Cooper GS Bynum ML Somers EC. Recent insights in the epidemiology of autoimmune diseases: improved prevalence estimates and understanding of clustering of diseases. J Autoimmun. (2009) 33:197–207. doi: 10.1016/j.jaut.2009.09.008, PMID: 19819109 PMC2783422

[5] Moutsopoulos HM. Autoimmune rheumatic diseases: One or many diseases? J Transl Autoimmun. (2021) 4:100129. doi: 10.1016/j.jtauto.2021.100129, PMID: 35005593 PMC8716565

[6] Szekanecz Z McInnes IB Schett G Szamosi S Benkő S Szűcs G. Autoinflammation and autoimmunity across rheumatic and musculoskeletal diseases. Nat Rev Rheumatol. (2021) 17:585–95. doi: 10.1038/s41584-021-00652-9, PMID: 34341562

[7] Islam MM Satici MO Eroglu SE. Unraveling the clinical significance and prognostic value of the neutrophil-to-lymphocyte ratio, platelet-to-lymphocyte ratio, systemic immune-inflammation index, systemic inflammation response index, and delta neutrophil index: An extensive literature review. Turk J Emerg Med. (2024) 24:8–19. doi: 10.4103/tjem.tjem_198_23, PMID: 38343523 PMC10852137

[8] Tian BW Yang YF Yang CC Yan LJ Ding ZN Liu H. Systemic immune-inflammation index predicts prognosis of cancer immunotherapy: systemic review and meta-analysis. Immunotherapy. (2022) 14:1481–96. doi: 10.2217/imt-2022-0133, PMID: 36537255

[9] Ye Z Hu T Wang J Xiao R Liao X Liu M. Systemic immune-inflammation index as a potential biomarker of cardiovascular diseases: A systematic review and meta-analysis. Front Cardiovasc Med. (2022) 9:933913. doi: 10.3389/fcvm.2022.933913, PMID: 36003917 PMC9393310

[10] Kim Y Choi H Jung SM Song JJ Park YB Lee SW. Systemic immune-inflammation index could estimate the cross-sectional high activity and the poor outcomes in immunosuppressive drug-naïve patients with antineutrophil cytoplasmic antibody-associated vasculitis. Nephrol (Carlton). (2019) 24:711–7. doi: 10.1111/nep.13491, PMID: 30203901

[11] Hu B Yang X-R Xu Y Sun Y-F Sun C Guo W. Systemic immune-inflammation index predicts prognosis of patients after curative resection for hepatocellular carcinoma. Clin Cancer Res. (2014) 20:6212–22. doi: 10.1158/1078-0432.CCR-14-0442, PMID: 25271081

[12] He L Xie X Xue J Xie H Zhang Y. Association of the systemic immune-inflammation index with all-cause mortality in patients with arteriosclerotic cardiovascular disease. Front Cardiovasc Med. (2022) 9:952953. doi: 10.3389/fcvm.2022.952953, PMID: 36172591 PMC9510918

[13] Li H Wu X Bai Y Wei W Li G Fu M. Physical activity attenuates the associations of systemic immune-inflammation index with total and cause-specific mortality among middle-aged and older populations. Sci Rep. (2021) 11:12532. doi: 10.1038/s41598-021-91324-x, PMID: 34131164 PMC8206152

[14] Guo W Song Y Sun Y Du H Cai Y You Q. Systemic immune-inflammation index is associated with diabetic kidney disease in Type 2 diabetes mellitus patients: Evidence from NHANES 2011-2018. Front Endocrinol (Lausanne). (2022) 13:1071465. doi: 10.3389/fendo.2022.1071465, PMID: 36561561 PMC9763451

[15] Tang Y Peng B Liu J Liu Z Xia Y Geng B. Systemic immune-inflammation index and bone mineral density in postmenopausal women: A cross-sectional study of the national health and nutrition examination survey (NHANES) 2007-2018. Front Immunol. (2022) 13:975400. doi: 10.3389/fimmu.2022.975400, PMID: 36159805 PMC9493473

[16] Wang J Zhou D Dai Z Li X. Association between systemic immune-inflammation index and diabetic depression. Clin Interv Aging. (2021) 16:97–105. doi: 10.2147/CIA.S285000, PMID: 33469277 PMC7810592

[17] Pritzker KPH. Blood-based biomarkers of chronic inflammation. Expert Rev Mol Diagn. (2023) 23:495–504. doi: 10.1080/14737159.2023.2215928, PMID: 37199146

[18] Tan KW Chong SZ Wong FH Evrard M Tan SM Keeble J. Neutrophils contribute to inflammatory lymphangiogenesis by increasing VEGF-A bioavailability and secreting VEGF-D. Blood. (2013) 122:3666–77. doi: 10.1182/blood-2012-11-466532, PMID: 24113869

[19] Wright HL Moots RJ Edwards SW. The multifactorial role of neutrophils in rheumatoid arthritis. Nat Rev Rheumatol. (2014) 10:593–601. doi: 10.1038/nrrheum.2014.80, PMID: 24914698

[20] Weyand CM Goronzy JJ. Immunometabolism in the development of rheumatoid arthritis. Immunol Rev. (2020) 294:177–87. doi: 10.1111/imr.12838, PMID: 31984519 PMC7047523

[21] Kaplan MJ. Neutrophils in the pathogenesis and manifestations of SLE. Nat Rev Rheumatol. (2011) 7:691–9. doi: 10.1038/nrrheum.2011.132, PMID: 21947176 PMC3243068

[22] Wehr P Purvis H Law SC Thomas R. Dendritic cells, T cells and their interaction in rheumatoid arthritis. Clin Exp Immunol. (2019) 196:12–27. doi: 10.1111/cei.13256, PMID: 30589082 PMC6422662

[23] Chemin K Gerstner C Malmström V. Effector functions of CD4+ T cells at the site of local autoimmune inflammation-lessons from rheumatoid arthritis. Front Immunol. (2019) 10:353. doi: 10.3389/fimmu.2019.00353, PMID: 30915067 PMC6422991

[24] McInnes IB Schett G. The pathogenesis of rheumatoid arthritis. N Engl J Med. (2011) 365:2205–19. doi: 10.1056/NEJMra1004965, PMID: 22150039

[25] Zhang Y Wang J Fang Y Liang W Lei L Wang J. IFN-α affects Th17/Treg cell balance through c-Maf and associated with the progression of EBV- SLE. Mol Immunol. (2024) 171:22–35. doi: 10.1016/j.molimm.2024.05.003, PMID: 38749236

[26] Shan J Jin H Xu Y. T cell metabolism: A new perspective on th17/treg cell imbalance in systemic lupus erythematosus. Front Immunol. (2020) 11:1027. doi: 10.3389/fimmu.2020.01027, PMID: 32528480 PMC7257669

[27] Wang D Huang S Yuan X Liang J Xu R Yao G. The regulation of the Treg/Th17 balance by mesenchymal stem cells in human systemic lupus erythematosus. Cell Mol Immunol. (2017) 14:423–31. doi: 10.1038/cmi.2015.89, PMID: 26435067 PMC5423084

[28] Cloutier N Paré A Farndale RW Schumacher HR Nigrovic PA Lacroix S. Platelets can enhance vascular permeability. Blood. (2012) 120:1334–43. doi: 10.1182/blood-2012-02-413047, PMID: 22544703

[29] Boilard E Blanco P Nigrovic PA. Platelets: active players in the pathogenesis of arthritis and SLE. Nat Rev Rheumatol. (2012) 8:534–42. doi: 10.1038/nrrheum.2012.118, PMID: 22868927

[30] Littlejohn EA Monrad SU. Early diagnosis and treatment of rheumatoid arthritis. Primary Care: Clinics Office Practice. (2018) 45:237–55. doi: 10.1016/j.pop.2018.02.010, PMID: 29759122

[31] Giannini D Antonucci M Petrelli F Bilia S Alunno A Puxeddu I. One year in review 2020: pathogenesis of rheumatoid arthritis. Clin Exp Rheumatol. (2020) 38:387–97. doi: 10.55563/clinexprheumatol/3uj1ng, PMID: 32324123

[32] Scherer HU Häupl T Burmester GR. The etiology of rheumatoid arthritis. J Autoimmun. (2020) 110:102400. doi: 10.1016/j.jaut.2019.102400, PMID: 31980337

[33] Weyand CM Goronzy JJ. The immunology of rheumatoid arthritis. Nat Immunol. (2021) 22:10–8. doi: 10.1038/s41590-020-00816-x, PMID: 33257900 PMC8557973

[34] Li NL Zhang DQ Zhou KY Cartman A Leroux JY Poole AR. Isolation and characteristics of autoreactive T cells specific to aggrecan G1 domain from rheumatoid arthritis patients. Cell Res. (2000) 10:39–49. doi: 10.1038/sj.cr.7290034, PMID: 10765982

[35] Lu M Gong L Huang C Ye M Wang H Liu Y. Analysis of clinical characteristics of connective tissue disease-associated interstitial lung disease in 161 patients: A retrospective study. Int J Gen Med. (2022) 15:8617–25. doi: 10.2147/IJGM.S391146, PMID: 36545245 PMC9762753

[36] Jang S Kwon EJ Lee JJ. Rheumatoid arthritis: pathogenic roles of diverse immune cells. Int J Mol Sci. (2022) 23(2):905. doi: 10.3390/ijms23020905, PMID: 35055087 PMC8780115

[37] El-Tanbouly GS Abdelrahman RS. Novel anti-arthritic mechanisms of trans-cinnamaldehyde against complete Freund’s adjuvant-induced arthritis in mice: involvement of NF-кB/TNF-α and IL-6/IL-23/IL-17 pathways in the immuno-inflammatory responses. Inflammopharmacology. (2022) 30:1769–80. doi: 10.1007/s10787-022-01005-y, PMID: PMC9499911 35648328

[38] Satis S. New inflammatory marker associated with disease activity in rheumatoid arthritis: the systemic immune-inflammation index. Curr Health Sci J. (2021) 47(4):553–7. doi: 10.12865/chsj.47.04.11, PMID: 35444819 PMC8987472

[39] Choe J-Y Lee CU Kim S-K. Association between novel hematological indices and measures of disease activity in patients with rheumatoid arthritis. Medicina. (2023) 59(1):117. doi: 10.3390/medicina59010117, PMID: 36676741 PMC9862645

[40] Liu B Wang J Li YY Li KP Zhang Q. The association between systemic immune-inflammation index and rheumatoid arthritis: evidence from NHANES 1999-2018. Arthritis Res Ther. (2023) 25:34. doi: 10.1186/s13075-023-03018-6, PMID: 36871051 PMC9985219

[41] Bai J Tian Y Wang F Dang J Wang H. Role of systemic immune-inflammation index (SII) in assessing clinical efficacy of TNF-α inhibitors for rheumatoid arthritis. Am J Transl Res. (2023) 15:6524–33., PMID: 38074808 PMC10703666

[42] Zhao J Jia Y Zeng L Huang H Liang G Hong K. Interplay of systemic immune-inflammation index and serum klotho levels: unveiling a new dimension in rheumatoid arthritis pathology. Int J Med Sci. (2024) 21:396–403. doi: 10.7150/ijms.89569, PMID: 38169796 PMC10758150

[43] Abraham CR Li A. Aging-suppressor Klotho: Prospects in diagnostics and therapeutics. Ageing Res Rev. (2022) 82:101766. doi: 10.1016/j.arr.2022.101766, PMID: 36283617

[44] Cheikhi A Barchowsky A Sahu A Shinde SN Pius A Clemens ZJ. Klotho: an elephant in aging research. J Gerontol A Biol Sci Med Sci. (2019) 74:1031–42. doi: 10.1093/gerona/glz061, PMID: 30843026 PMC7330474

[45] Fu Y Cao J Wei X Ge Y Su Z Yu D. Klotho alleviates contrast-induced acute kidney injury by suppressing oxidative stress, inflammation, and NF-KappaB/NLRP3-mediated pyroptosis. Int Immunopharmacol. (2023) 118:110105. doi: 10.1016/j.intimp.2023.110105, PMID: 37018977

[46] Kaszubowska L Foerster J Kaczor JJ Karnia MJ Kmieć Z. Anti-inflammatory klotho protein serum concentration correlates with interferon gamma expression related to the cellular activity of both NKT-like and T cells in the process of human aging. Int J Mol Sci. (2023) 24(9):8393. doi: 10.3390/ijms24098393, PMID: 37176100 PMC10179552

[47] Han YY Celedón JC Forno E. Serum α-Klotho level, lung function, airflow obstruction and inflammatory markers in US adults. ERJ Open Res. (2023) 9(6):00471. doi: 10.1183/23120541.00471-2023, PMID: 37936898 PMC10626412

[48] Wu SE Chen WL. Soluble klotho as an effective biomarker to characterize inflammatory states. Ann Med. (2022) 54:1520–9. doi: 10.1080/07853890.2022.2077428, PMID: 35603960 PMC9132391

[49] Singh A Behl T Sehgal A Singh S Sharma N Naved T. Mechanistic insights into the role of B cells in rheumatoid arthritis. Int Immunopharmacol. (2021) 99:108078. doi: 10.1016/j.intimp.2021.108078, PMID: 34426116

[50] Basaran PO Dogan M. The relationship between disease activity with pan-immune-inflammatory value and systemic immune-inflammation index in rheumatoid arthritis. Med (Baltimore). (2024) 103:e37230. doi: 10.1097/MD.0000000000037230, PMID: 38428850 PMC10906570

[51] Tutan D Dogan AG. Pan-immune-inflammation index as a biomarker for rheumatoid arthritis progression and diagnosis. Cureus. (2023) 15:e46609. doi: 10.7759/cureus.46609, PMID: 37808603 PMC10558813

[52] Xu Y He H Zang Y Yu Z Hu H Cui J. Systemic inflammation response index (SIRI) as a novel biomarker in patients with rheumatoid arthritis: a multi-center retrospective study. Clin Rheumatol. (2022) 41:1989–2000. doi: 10.1007/s10067-022-06122-1, PMID: 35266094

[53] Carron P De Craemer AS Van den Bosch F. Peripheral spondyloarthritis: a neglected entity-state of the art. RMD Open. (2020) 6(1):e001136. doi: 10.1136/rmdopen-2019-001136, PMID: 32385142 PMC7299516

[54] Rachid B El Zorkany B Youseif E Tikly M. Early diagnosis and treatment of ankylosing spondylitis in Africa and the Middle East. Clin Rheumatol. (2012) 31:1633–9. doi: 10.1007/s10067-012-2058-5, PMID: 22903740

[55] Hioki T Komine M Ohtsuki M. Diagnosis and intervention in early psoriatic arthritis. J Clin Med. (2022) 11(7):2051. doi: 10.3390/jcm11072051, PMID: 35407659 PMC8999837

[56] Seng JJB Kwan YH Low LL Thumboo J Fong WSW. Role of neutrophil to lymphocyte ratio (NLR), platelet to lymphocyte ratio (PLR) and mean platelet volume (MPV) in assessing disease control in Asian patients with axial spondyloarthritis. Biomarkers. (2018) 23:335–8. doi: 10.1080/1354750X.2018.1425916, PMID: 29307233

[57] Targonska-Stepniak B Grzechnik K. The usefulness of cellular immune inflammation markers and ultrasound evaluation in the assessment of disease activity in patients with spondyloarthritis. J Clin Med. (2023) 12(17):5463. doi: 10.3390/jcm12175463, PMID: 37685529 PMC10488089

[58] Wu J Yan L Chai K. Systemic immune-inflammation index is associated with disease activity in patients with ankylosing spondylitis. J Clin Lab Anal. (2021) 35:e23964. doi: 10.1002/jcla.23964, PMID: 34418163 PMC8418483

[59] Tashiro T Sawada Y. Psoriasis and systemic inflammatory disorders. Int J Mol Sci. (2022) 23(8):4457. doi: 10.3390/ijms23084457, PMID: 35457278 PMC9028262

[60] Wu JJ Kavanaugh A Lebwohl MG Gniadecki R Merola JF. Psoriasis and metabolic syndrome: implications for the management and treatment of psoriasis. J Eur Acad Dermatol Venereol. (2022) 36:797–806. doi: 10.1111/jdv.18044, PMID: 35238067 PMC9313585

[61] Tamer F Edek YC Aksakal AB. Effect of treatment with biologic agents on the novel inflammatory biomarkers systemic immune inflammation index and systemic inflammation response index for psoriasis. Dermatol Pract Concept. (2024) 14(1):e2024065. doi: 10.5826/dpc.1401a65, PMID: 38364414 PMC10868908

[62] Yorulmaz A Hayran Y Akpinar U Yalcin B. Systemic immune-inflammation index (SII) predicts increased severity in psoriasis and psoriatic arthritis. Curr Health Sci J. (2020) 46(4):352–7. doi: 10.12865/chsj.46.04.05, PMID: 33717509 PMC7948012

[63] Kelesoglu Dincer AB Sezer S. Systemic immune inflammation index as a reliable disease activity marker in psoriatic arthritis. J Coll Physicians Surg Pak. (2022) 32:773–8. doi: 10.29271/jcpsp.2022.06.773, PMID: 35686411

[64] Aringer M Johnson SR. Systemic lupus erythematosus classification and diagnosis. Rheum Dis Clin North Am. (2021) 47:501–11. doi: 10.1016/j.rdc.2021.04.011, PMID: 34215376

[65] Graham JJ Longhi MS Heneghan MA. T helper cell immunity in pregnancy and influence on autoimmune disease progression. J Autoimmun. (2021) 121:102651. doi: 10.1016/j.jaut.2021.102651, PMID: 34020252 PMC8221281

[66] Jeleniewicz R Suszek D Majdan M. Clinical picture of late-onset systemic lupus erythematosus in a group of Polish patients. Pol Arch Med Wewn. (2015) 125:538–44. doi: 10.20452/pamw.2963, PMID: 26075796

[67] Clarke AE Weinstein A Piscitello A Heer A Chandra T Doshi S. Evaluation of the economic benefit of earlier systemic lupus erythematosus (SLE) diagnosis using a multivariate assay panel (MAP). ACR Open Rheumatol. (2020) 2:629–39. doi: 10.1002/acr2.11177, PMID: 33044050 PMC7672303

[68] Reshetnyak T Nurbaeva K. The role of neutrophil extracellular traps (NETs) in the pathogenesis of systemic lupus erythematosus and antiphospholipid syndrome. Int J Mol Sci. (2023) 24(17):13581. doi: 10.3390/ijms241713581, PMID: 37686381 PMC10487763

[69] Moysidou E Christodoulou M Lioulios G Stai S Karamitsos T Dimitroulas T. Lymphocytes change their phenotype and function in systemic lupus erythematosus and lupus nephritis. Int J Mol Sci. (2024) 25(20):10905. doi: 10.3390/ijms252010905, PMID: 39456692 PMC11508046

[70] Scherlinger M Sisirak V Richez C Lazaro E Duffau P Blanco P. New insights on platelets and platelet-derived microparticles in systemic lupus erythematosus. Curr Rheumatol Rep. (2017) 19:48. doi: 10.1007/s11926-017-0678-0, PMID: 28718063

[71] Akdogan MR Melikoglu MA. A potential biomarker of disease activity in systemic lupus erythematosus, systemic immune-inflammation index. North Clin Istanb. (2024) 11:115–9. doi: 10.14744/nci.2023.90132, PMID: 38757099 PMC11095327

[72] Predescu OR Vreju AF Dinescu SC Florescu A Bita CE Musetescu AE. Potential biomarkers for disease activity in systemic lupus erythematosus. Curr Health Sci J. (2024) 50(3):347–52. doi: 10.12865/chsj.50.03.01, PMID: 39574502 PMC11578361

[73] Baykal G Vazgeçer EO Sözeri B. Assessment of hematologic indices for diagnosis in juvenile systemic lupus erythematosus. Rheumatology. (2024) 62(2):74–82. doi: 10.5114/reum/186826, PMID: 38799776 PMC11114125

[74] Ergun MC Aktas E Sahin AT Iyisoy MS Alsancak Y Tunc R. Systemic immune-inflammation index as a potential biomarker for assessing disease activity and predicting proteinuria development in systemic lupus erythematosus. Cureus. (2024) 16:e63401. doi: 10.7759/cureus.63401, PMID: 39070439 PMC11283863

[75] Yang CH Wang XY Zhang YH Ding N. SIRI and SII as potential biomarkers of disease activity and lupus nephritis in systemic lupus erythematosus. Front Immunol. (2025) 16:1530534. doi: 10.3389/fimmu.2025.1530534, PMID: 39958362 PMC11825474

[76] Ozdemir A Baran E Kutu M Celik S Yilmaz M. Could systemic immune inflammation index be a new parameter for diagnosis and disease activity assessment in systemic lupus erythematosus? Int Urol Nephrol. (2023) 55:211–6. doi: 10.1007/s11255-022-03320-3, PMID: 35918626

[77] Sahin R Tanacan A Serbetci H Agaoglu Z Karagoz B Haksever M. The role of first-trimester NLR (neutrophil to lymphocyte ratio), systemic immune-inflammation index (SII), and, systemic immune-response index (SIRI) in the prediction of composite adverse outcomes in pregnant women with systemic lupus erythematosus. J Reprod Immunol. (2023) 158:103978. doi: 10.1016/j.jri.2023.103978, PMID: 37329867

[78] Gambichler T Numanovic Z Apel I Hessam S Susok L Xenofon B. Do novel inflammation biomarkers arising from routine complete blood count play a role in patients with systemic lupus erythematosus? Lupus. (2024) 33:1556–61. doi: 10.1177/09612033241295865, PMID: 39435639

[79] Taha SI Samaan SF Ibrahim RA Moustafa NM El-Sehsah EM Youssef MK. Can complete blood count picture tell us more about the activity of rheumatological diseases? Clin Med Insights: Arthritis Musculoskeletal Disord. (2022) 15:11795441221089182. doi: 10.1177/11795441221089182, PMID: 35481333 PMC9036329

[80] Moretti M Treppo E Monti S La Rocca G Del Frate G Delvino P. Systemic vasculitis: one year in review 2023. Clin Exp Rheumatol. (2023) 41:765–73. doi: 10.55563/clinexprheumatol/zf4daj, PMID: 37073639

[81] Maz M Chung SA Abril A Langford CA Gorelik M Guyatt G. American college of rheumatology/Vasculitis foundation guideline for the management of giant cell arteritis and takayasu arteritis. Arthritis Rheumatol. (2021) 73:1349–65. doi: 10.1002/art.41774, PMID: 34235884 PMC12344528

[82] McCrindle BW Rowley AH Newburger JW Burns JC Bolger AF Gewitz M. Diagnosis, treatment, and long-term management of kawasaki disease: A scientific statement for health professionals from the american heart association. Circulation. (2017) 135:e927–e99. doi: 10.1161/CIR.0000000000000484, PMID: 28356445

[83] Chen JB Tang R Zhong Y Zhou YO Zuo X Luo H. Systemic immune-inflammation index predicts a reduced risk of end-stage renal disease in Chinese patients with myeloperoxidase-anti-neutrophil cytoplasmic antibody-associated vasculitis: A retrospective observational study. Exp Ther Med. (2021) 22:989. doi: 10.3892/etm.2021.10421, PMID: 34345271 PMC8311255

[84] Tanacan E Dincer D Erdogan FG Gurler A. A cutoff value for the Systemic Immune-Inflammation Index in determining activity of Behcet disease. Clin Exp Dermatol. (2021) 46:286–91. doi: 10.1111/ced.14432, PMID: 32869876

[85] Mentesoglu D Atakan N. The association between Behcet disease activity and elevated systemic immune-inflammation index: A retrospective observational study in a tertiary care hospital. Natl Med J India. (2024) 37:74–8. doi: 10.25259/NMJI_212_2022, PMID: 39222532

[86] Ulusoy B Armağan B Erdoğan EK Maraş Y Doğan İ Orhan K. Novel inflammatory biomarkers for discriminating vascular involvement in Behçet’s syndrome: evaluating SII, SIRI, and NPAR. biomark Med. (2025) 19:149–56. doi: 10.1080/17520363.2025.2473312, PMID: 40024670 PMC11916354

[87] Huang T Peng Q Zhang Y Zhu Z Fan X. The Systemic Immune-Inflammation Index (SII) and coronary artery lesions in Kawasaki disease. Clin Exp Med. (2024) 24:4. doi: 10.1007/s10238-023-01265-0, PMID: 38231301 PMC10794328

[88] Yalcinkaya R Oz FN Durmus SY Fettah A Kaman A Teke TA. Is there a role for laboratory parameters in predicting coronary artery involvement in kawasaki disease? Klin Padiatr. (2022) 234:382–7. doi: 10.1055/a-1816-6754, PMID: 35785802

[89] Liu J Ye B Su D Qin S Zhao W Pang Y. Evaluation of laboratory predictors for intravenous immunoglobulin resistance and coronary artery aneurysm in Kawasaki Disease before and after therapy. Clin Rheumatol. (2023) 42:167–77. doi: 10.1007/s10067-022-06366-x, PMID: 36129563 PMC9491265

[90] Yi C Zhou YN Guo J Chen J She X. Novel predictors of intravenous immunoglobulin resistance in patients with Kawasaki disease: a retrospective study. Front Immunol. (2024) 15:1399150. doi: 10.3389/fimmu.2024.1399150, PMID: 39040113 PMC11260624

[91] Dong M Shi Y Yang J Zhou Q Lian Y Wang D. Prognostic and clinicopathological significance of systemic immune-inflammation index in colorectal cancer: a meta-analysis. Ther Adv Med Oncol. (2020) 12:1758835920937425. doi: 10.1177/1758835920937425, PMID: 32699557 PMC7357045

[92] Wu Y Zhao J Wang Z Liu D Tian C Ye B. Association of systemic inflammatory markers and tertiary lymphoid structure with pathological complete response in gastric cancer patients receiving preoperative treatment: a retrospective cohort study. Int J Surg. (2023) 109:4151–61. doi: 10.1097/JS9.0000000000000741, PMID: 38259000 PMC10720847

[93] Huai Q Luo C Song P Bie F Bai G Li Y. Peripheral blood inflammatory biomarkers dynamics reflect treatment response and predict prognosis in non-small cell lung cancer patients with neoadjuvant immunotherapy. Cancer Sci. (2023) 114:4484–98. doi: 10.1111/cas.15964, PMID: 37731264 PMC10728017

[94] Menyhart O Fekete JT Gyorffy B. Inflammation and colorectal cancer: A meta-analysis of the prognostic significance of the systemic immune-inflammation index (SII) and the systemic inflammation response index (SIRI). Int J Mol Sci. (2024) 25(15):8441. doi: 10.3390/ijms25158441, PMID: 39126008 PMC11312822

[95] Nost TH Alcala K Urbarova I Byrne KS Guida F Sandanger TM. Systemic inflammation markers and cancer incidence in the UK Biobank. Eur J Epidemiol. (2021) 36:841–8. doi: 10.1007/s10654-021-00752-6, PMID: 34036468 PMC8416852

[96] Jiang D Bian T Shen Y Huang Z. Association between admission systemic immune-inflammation index and mortality in critically ill patients with sepsis: a retrospective cohort study based on MIMIC-IV database. Clin Exp Med. (2023) 23:3641–50. doi: 10.1007/s10238-023-01029-w, PMID: 36930382 PMC10022570

[97] Bolos OC Sorca BV Rusu LC Tapalaga G. Comparative Assessment of the qSOFA, SII, dNLR, and OISS Infection Severity Scores in Diabetic Versus Non-Diabetic Patients with Odontogenic Infections. Biomedicines. (2024) 12(12):2712. doi: 10.3390/biomedicines12122712, PMID: 39767619 PMC11672987

[98] Wang RH Wen WX Jiang ZP Du ZP Ma ZH Lu AL. The clinical value of neutrophil-to-lymphocyte ratio (NLR), systemic immune-inflammation index (SII), platelet-to-lymphocyte ratio (PLR) and systemic inflammation response index (SIRI) for predicting the occurrence and severity of pneumonia in patients with intracerebral hemorrhage. Front Immunol. (2023) 14:1115031. doi: 10.3389/fimmu.2023.1115031, PMID: 36860868 PMC9969881

[99] Zhao G Chen Y Gu Y Xia X. The clinical value of nutritional and inflammatory indicators in predicting pneumonia among patients with intracerebral hemorrhage. Sci Rep. (2024) 14:16171. doi: 10.1038/s41598-024-67227-y, PMID: 39003396 PMC11246476

[100] Li M Nie Y Yang Z. The association between systemic immune-inflammation index (SII) and early nosocomial infections after cardiopulmonary bypass surgery in children with congenital heart disease. BMC Cardiovasc Disord. (2024) 24:698. doi: 10.1186/s12872-024-04378-w, PMID: 39633275 PMC11619183

